# S1PR_4_ deficiency results in reduced germinal center formation but only marginally affects antibody production

**DOI:** 10.3389/fimmu.2022.1053490

**Published:** 2022-12-02

**Authors:** Janik Riese, Celine Hähnel, Jonas Menz, Maurice Hannemann, Aydar Khabipov, Felix Lührs, Tobias Schulze

**Affiliations:** Experimental Surgical Research Laboratory, Department of General Surgery, Visceral, Thoracic and Vascular Surgery, Universitätsmedizin Greifswald, Greifswald, Germany

**Keywords:** B cells, spleen, germinal center reaction, antibody production, abdominal sepsis, Sphingosine-1-phosphate

## Abstract

**Introduction:**

Splenic B cells exhibit a high expression of the G protein-coupled sphingosine-1-phosphate (S1P) receptor type 4 (S1PR_4_). Little is known about the functional relevance of S1PR_4_ expression on those cells.

**Methods:**

In this study, S1PR_4_-deficient mice were used to study the role of S1PR_4_-mediated S1P signaling in B cell motility *in vitro* and for the maintenance of the splenic architecture under steady state conditions as well as in polymicrobial abdominal sepsis *in vivo*. Finally, the impact of S1PR_4_ deficiency on antibody production after immunization with T cell dependent antigens was assessed.

**Results:**

Loss of S1PR_4_ resulted in minor alterations of the splenic architecture concerning the presence of B cell follicles. After sepsis induction, the germinal center response was severely impaired in S1PR_4_-deficient animals. Splenic B cells showed reduced motility in the absence of S1PR_4_. However, titres of specific antibodies showed only minor reductions in S1PR_4_-deficient animals.

**Discussion:**

These observations suggest that S1P signaling mediated by S1PR_4_ modifies chemokine-induced splenic B cell chemotaxis, thus modulating splenic microarchitecture, GC formation and T-cell dependent antibody production.

## Introduction

The formation of an effective humoral immune response is dependent on intricate interactions between B cells and other immune cell types. These interactions are enabled by the precise localization of these various immune cells in their respective niches (1). The spleen is a highly compartmentalized organ, and its intact histological structure is a prerequisite for the establishment of an effective humoral immune response (2, 3). The positioning of follicular B cells and other immune cells within the spleen is a highly dynamic process orchestrated by a complex network of chemokines and cytokines, but also by other secreted molecules like sphingosine-1-phosphate (S1P) (1, 4–9). S1P is a bioactive lipid that exhibits a steep gradient between high vascular concentrations and low presence in the surrounding interstitium (10). S1P binds to a family of five membrane bound S1P receptors S1PR_1–5_. While S1PR_1–3_ are nearly ubiquitously expressed, S1PR_4_ expression is restricted to cells of hematopoietic origin (11). By blocking S1P signaling with a functional antagonist of S1P receptors S1PR_1_ and S1PR_3–5_, Han et al. revealed a role of S1P signaling in the germinal center reaction (12). However, the identity of the S1P receptor mediating this effect remains unknown.

In a previous study, we have reported a reduced migration of LPS stimulated peritoneal B cells from the abdominal cavity into splenic follicles (13). S1PR_4_ expression is a common feature of various splenic B cell populations. In the present study, we sought to characterize the impact of S1PR_4_-mediated signaling on the splenic architecture and the development of the B cell response during systemic infections.

## Materials and methods

### Mice

Mice deficient for the S1PR_4_ receptor ( $s1pr_{4}^{-/-}$ ) were bred on a C57BL/6J background under specific pathogen-free (SPF) conditions (14). SPF female C57BL/6J mice (*wt*) were purchased from Charles River (Sulzfeld, Germany) and used for control purposes. All mice in the experiments were of female sex and aged between 10 and 14 weeks. All animal care practices and experimental procedures were performed in accordance with the German animal protection law (*TierSchuG*) and controlled by the appropriate veterinary government authority.

### Colon ascendens stent peritonitis (CASP)

A model with close resemblance to clinical sepsis was chosen for sepsis induction. CASP surgery induces a polymicrobial, abdominal sepsis. The surgery was performed as described (15). Briefly, anaesthetized mice were placed in a supine position and the peritoneum was opened along a 1 cm midline incision along the linea alba. A small plastic stent (18 G needle; BD Bioscience, Heidelberg, Germany) was inserted into the antimesenteric wall of the cecum 1 cm distal to the ileocecal valve and fixed using a 7/0 suture. Before closure of the abdominal wall, fluid resuscitation was performed *via* 0.5 mL isosmotic NaCl solution intraperitoneally. The peritoneum and adjacent skin were closed with a two-layer closure.

### Fluorescence microscopy

For organ harvesting, mice were anaesthetized and then sacrificed by cervical dislocation. Spleens were removed, embedded in TissueTek (Sakura Finetek Europe B.V., Alphen aan den Rijn, Netherlands) and immediately snap-frozen in nitrogen-cooled isopentane. Sections for microscopical analysis were cut at a cryostat and afterwards dried for 24 hours with subsequent fixation in acetone. Non-specific and biotin binding sites were blocked with phosphate buffered saline + 10% fetal calf serum or Dako Biotin Blocking System (Dako North America Inc., Carpinteria, CA, USA), respectively. After appropriate washing cycles, sections were incubated with specific antibodies. Cell nuclei were stained with DAPI (Molecular Probes, Eugene, OR, USA) or Draq5™ (Biolegend, San Diego, CA, USA). The following antibodies and conjugates were used in the experiments in appropriate combinations: anti-B220-FITC (clone: RA3-6B2; Miltenyi Biotec), anti-B220-AlexaFluor647 (clone: RA3-6B2; Miltenyi Biotec), anti-CD4-BV421 (clone: GK1.5; BioLegend), biotin conjugated GL-7 antibody (eBioscience), anti-IgD-biotin (clone: MD78Z; Southern Biotech), anti-IgM-FITC (clone: II/41; Invitrogen), anti-MAdCAM1-AlexaFluor488 (clone: MECA-367; BioLegend), anti-CD35-biotin (clone: 2A0Q1; Invitrogen), anti-CD86-BV421 (clone: GL-1; BioLegend), PNA-FITC (Vector Laboratories). For each experiment the same exposure time was used for all images. The open-source software QuPath was used for down-stream analysis of area measurements and cell identification (16). All microscopic images were quantified using the cell classification algorithm implemented in QuPath. MZ B cell oscillation was quantified using QuPath for automated cell identification. Follicle border points were identified using Jarvis’ convex hull algorithm and continuously computed *via* Fourier transformation. Cell centroid coordinates were assigned to respective zones with an automated python algorithm.

### Live cell imaging

Live cell imaging of splenic B cells was performed using a BZ-9000E fluorescence microscope with incubation chamber (Keyence, Osaka, Japan). Splenic B cells were isolated and briefly stained with carboxyfluorescein succinimidyl ester (CFSE; BioLegend) according the manufacturers instructions. Afterwards cells were resuspended in RPMI-1640 medium with GlutaMAX™ supplement (Thermo Fisher Scientific), HEPES buffer, fetal calf serum (75 nM S1P) and 500 nM CXCL13 with carrier (Bio-Techne GmbH, Wiesbaden, Germany). After incubation for 20 minutes, a volume of 500 µL was transferred to 8-well µ-chamber slides (ibidi, Gräfelfing, Germany). Chamber slides were then placed inside of the incubator (37 °C; 5% CO_2_; 95% humidity) and incubated for an additional 30 minutes. Time-lapse images were captured every 30 seconds for 50 minutes. Final datasets were analyzed using the TrackMate plugin of ImageJ (17, 18). For chemotaxis assays, migration medium was thickened with agarose as previously described (19).

### Flow cytometry

Single cell suspensions of freshly harvested spleens were prepared using 40-µm-CellStrainer (Sarstedt). Red blood cells were lysed using RBC lysis buffer according to the instructions of the manufacturer (BioLegend). Non-specific binding sites were blocked with unconjugated anti-FcII/III (anti-CD16/32; BD Pharmingen, Heidelberg, Germany). The following antibodies and conjugates were used in the experiments in appropriate combinations: anti-CD19-APC (clone: 1D3; BD Pharmingen), anti-CD21-FITC (clone: 76G; BD Pharmingen), anti-CD23-PE (clone: B3B4; BD Pharmingen), anti-IgM-APC (clone: LO-MM-9; Invitrogen). Stained cells were analyzed using a BD LSR II Flow Cytometer (BD Biosciences) and evaluated with FlowJo software (Version 10; LLC, Ashland, OR, USA).

### Analysis of T cell dependent antibody production

Mice were immunized by intraperitoneal injection of 2 x 10^8^ sheep red blood cells (SRBCs; Merck, Darmstadt, Germany) dissolved in 200 µL isosmotic NaCl on days 0 and 15. At days 14, 21, 28 and 49, blood was drawn by retro-orbital puncture. For control purposes, blood of non-immunized mice was also analyzed. Plasma titers of specific anti-SRBC antibodies (IgG1, IgM) were determined the SBA clonotyping system (Southern Biotech, Birmingham, AL, USA) according to the instructions of the manufacturer. Optical density was measured at 405 nm with an ELISA reader (Power Wave X Select; BIO-TEK Instruments, Winooski, VT, USA).

### Statistical analysis

Statistical tests were performed with GraphPad Prism Software (Version 6.01; GraphPad Software Inc., La Jolla, CA, USA). All groups were tested for Gaussian distribution using Shapiro–Wilk test and homoscedasticity was assessed using Levene’s test. When groups were normally distributed, statistical analysis was performed by either a t-test or one-way analysis of variance (ANOVA) and Dunnett’s multiple comparison test; otherwise, either Mann−Whitney-U test or Kruskal−Wallis test with Dunn’s multiple comparison test was used. For antibody affinity tests, a repeated measure ANOVA was used. If assumption of homoscedasticity of groups was not met, Welch’s correction was applied. *p*-values< 0.05 were considered to be significant. Significance in the graphs is labeled as follows: * *p*< 0.05, ** *p*< 0.01, or *** *p*< 0.001.

## Results

### S1PR_4_ deficiency results in reduced splenic follicle size

**Figure 1:**
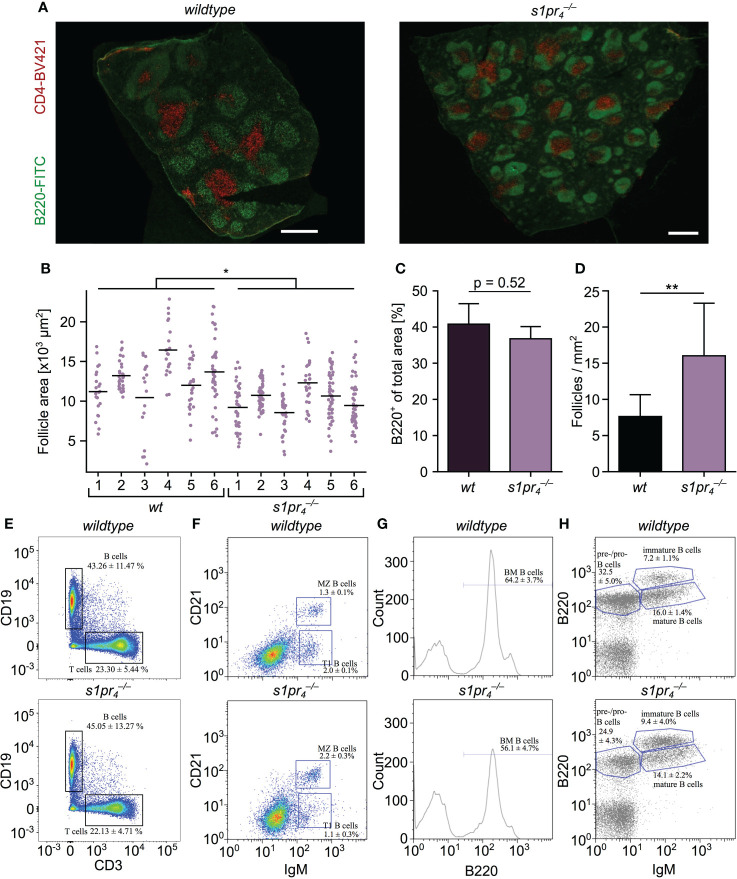
Analysis of the architecture and composition of the spleen. **(A)** Microscopic structure of spleens of wildtype (wt) and S1PR_4_-deficient ( $s1pr_{4}^{-/-}$ ) mice stained with anti-B220 (green) and anti-CD4 (red) to identify B zone and T zone, respectively. **(B–D)** Quantification of follicle size, density and B220 positive area. Values represent the mean + standard deviation of n = 6 animals per group. **(E, F)** Flow cytometric analysis of splenic lymphocyte populations. B cells: CD19^+^; T cells CD3^+^; MZ B cells: CD21^hi^ IgM^hi^; transitional type 1 B cells: CD21^lo^ IgM^hi^ (n = 5 per group). **(G)** Flow cytometric analysis of bone marrow cells stained with anti-B220 (n = 5 per group). **(H)** Analysis of B cell subpopulations in the bone marrow. Pre-/pro-B cells: B220^+^, IgM^–^; immature B cells: B220^int^, IgM^+^; mature B cells: B220^hi^, IgM^+^ (n = 5 per group). White scale bar equals 500 µm. *p< 0.05, **p< 0.01.

The splenic architecture of S1PR_4_-deficient ( $s1pr_{4}^{-/-}$ ) mice was evaluated by staining the B and T cell compartment with anti-B220 and anti-CD4 antibody, respectively. The structural composition of the white pulp of $s1pr_{4}^{-/-}$ mice was qualitatively similar to their wildtype (*wt*) counterparts. The spleen contained regular appearing B220^+^ follicles and CD4^+^ PALS (**Figure 1A**). However, quantification of the follicle area revealed a significant reduction of the follicle size in $s1pr_{4}^{-/-}$ animals when compared to their *wt* counterparts (**Figure 1B**). This reduction of follicle size did not result in a decrease of the total B220^+^ area (**Figure 1C**). An increase in follicle number did compensate for reduced follicle size (**Figure 1D**). Splenic B cell populations were analyzed in detail by flow cytometry. Total number of CD19^+^ B and CD3^+^ T cells did not differ between genotypes (**Figure 1E**; **Figure S1**). B cells were gated for transitional type 1 B cells (CD21^lo^, CD23^hi^, IgM^hi^), transitional type 2 B cells (CD21^hi^, CD23^+^, IgM^hi^), MZ B cells (CD21^hi^, CD23^+^, IgM^hi^) and mature follicular B cells (CD21^int^, CD23^+^, IgM^lo^). While transitional type 2 B cell numbers were similar in animals of both genotypes (data not shown), transitional type 1 B cell numbers were significantly reduced in $s1pr_{4}^{-/-}$ mice (1.1 ± 0.3% and 2.0 ± 0.1% in $s1pr_{4}^{-/-}$ and *wt* animals, respectively; *p*< 0.01) (**Figure 1F**; **Figure S2**). Also, MZ B cells were increased by almost 70% from 1.3 ± 0.1% in *wt* mice to 2.2 ± 0.3% in $s1pr_{4}^{-/-}$ mice. Since the splenic B cell pool is constantly replenished from the bone marrow (20) and transitional type 1 B cell numbers were reduced in the splenic compartment of $s1pr_{4}^{-/-}$ animals, flow cytometric analysis of the bone marrow B cell compartment was performed. Among the bone marrow lymphoid cells, B220^+^ B cell numbers were reduced in $s1pr_{4}^{-/-}$ mice compared to *wt* animals (56.1 ± 4.7% vs. 64.2 ± 3.7%, respectively; *p*< 0.01; **Figure 1G**). The B220^+^ IgM^–^ cell population that contains both pro- and pre-B cell stages was slightly but significantly reduced in the bone marrow of $s1pr_{4}^{-/-}$ mice (24.9 ± 4.3% vs. 32.5 ± 5.0%, respectively; *p*< 0.05; **Figure 1H**). Immature B cells identified as (B220^int^ IgM^+^) and mature B cells (B220^hi^ IgM^+^) were present in similar frequencies in $s1pr_{4}^{-/-}$ and *wt* animals.

### B cell responsiveness to CXCL13 is modified by S1PR_4_–mediated S1P-signaling

**Figure 2:**
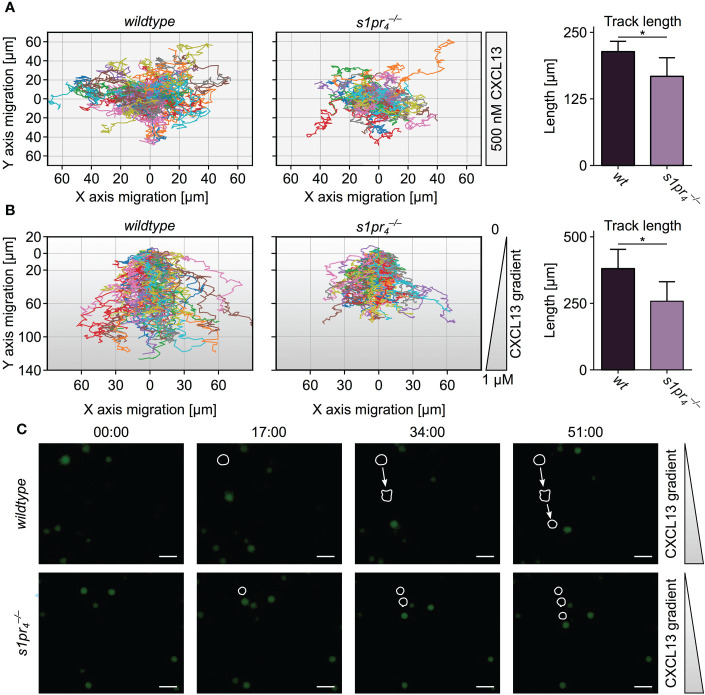
*Ex vivo* migration of splenic B cells in the presence of CXCL13. **(A)** Chemokinesis of CFSE-stained B cells in the presence of 500 nM CXCL13. **(B)** Chemotaxis of CFSE-stained B cells towards a CXCL13 gradient. Track length was quantified using TrackMate. Each color line represents the track of a specific cell. Representative image sequences are shown with exemplary cell tracks marked. *p< 0.05. Values represent mean + standard deviation of n = 5 animals per condition. **(C)** Time-lapse images representing the migration of individual cells toward a CXCL13 gradient. White scale bars equal 50 µm.

The formation of splenic compartments with their respective cell populations is dependent on spatio-temporal cell localization. Chemokine gradients, e.g. CXCL12 and CXCL13, tightly control cell localization *via* migrational processes (21). Thus, an altered responsiveness to these chemokines may account for the alterations in the splenic architecture observed in $s1pr_{4}^{-/-}$ animals. To test this hypothesis, the undirected (chemokinesis) and directed (chemotaxis) movement of splenic B cells in the presence of CXCL13 was analyzed *in vitro*. Using *ex vivo* live cell imaging, analysis of splenic B cell chemokinesis in the presence of CXCL13 showed a significant reduction in the velocity and the distance travelled of $s1pr_{4}^{-/-}$ B cells (**Figure 2A**). In the presence of a CXCL13 gradient, $s1pr_{4}^{-/-}$ B cells also showed a significant inhibition of the directed migration towards high CXCL13 concentrations when compared to splenic *wt* B cells (**Figures 2B, C**).

### Impaired germinal center formation in $s1pr_{4}^{–/–}$ mice after induction of abdominal sepsis

**Figure 3:**
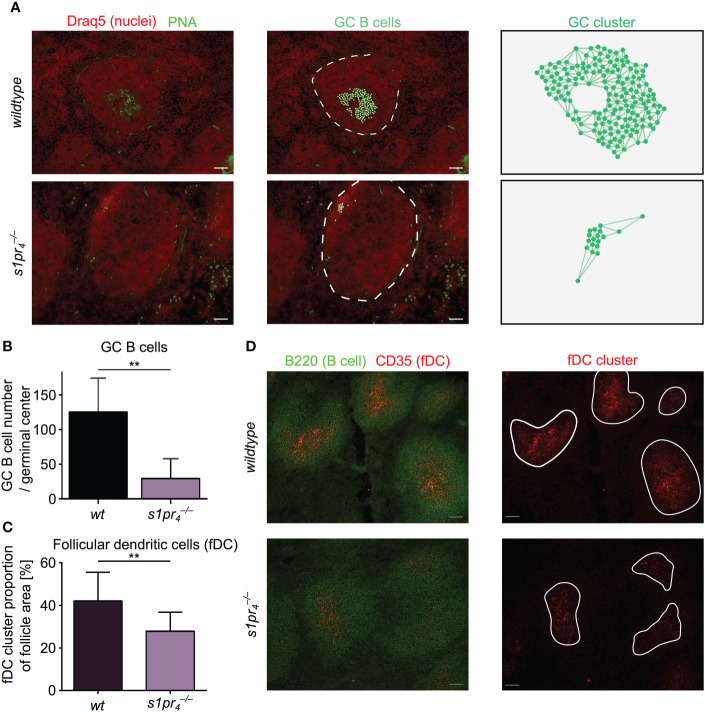
Morphological evaluation of germinal center dynamics after sepsis induction. **(A)** Seven days after sepsis induction, splenic sections were analyzed for the presence of germinal center (GC) using PNA (green). GC B cells were automatically segmented and cluster analysis was performed using QuPath. **(B)** GC from $s1pr_{4}^{-/-}$ mice were significantly smaller and showed excentric localization, when compared to wt counterparts. **(C, D)** Follicles were analyzed for the presence of CD35^+^ follicular dendritic cells (FDC). FDC clusters were present in both genotypes, although cluster size was decreased in $s1pr_{4}^{-/-}$ mice. All images representative for at least n = 3 animals per group. White scale bar equals 50 µm. **p < 0.01.

In the event of an infection, follicular B cells form germinal centers (GC) to progressively improve the affinity of the antibodies produced, thus providing the basis for an effective humoral immune response (22). Similarly, systemic bacterial infection and sepsis result in a strong GC response with an early increase of IgM and IgG antibody levels (23, 24). While lack of S1PR_4_ expression resulted only in moderate quantitative changes of the splenic structure under non-infectious conditions, we hypothesized that a systemic infectious challenge would result in an exacerbation of the structural consequences of S1PR_4_ deficiency. In order to test this hypothesis, GC formation was evaluated seven days after sepsis induction in a murine model of polymicrobial peritonitis, the Colon ascendens stent peritonitis (CASP) model (25). The size of GCs was greatly reduced in $s1pr_{4}^{-/-}$ animals compared to *wt* controls. This observation was validated using three different markers to identify GC B cells: PNA (**Figure 3A**), GL-7 and anti-CD86 (**Figure S3**). Germinal centers of *wt* mice contained significantly more GC B cells than germinal centers of $s1pr_{4}^{-/-}$ animals (**Figure 3B**).The formation and maintenance of GC depends on various factor, among them the presence of follicular dendritic cells (FDC). In various other models the lack of GC formation could be linked to insufficient niches containing FDCs (26). In order to exclude differences in the number of FDC clusters in the follicles of $s1pr_{4}^{-/-}$ and *wt* animals, spleens of septic mice from both genotypes were analyzed for the intrafollicular presence of CD35^+^ FDCs. Although follicles of both genotypes contained FDC clusters, the size of the individual FDC clusters was significantly reduced in $s1pr_{4}^{-/-}$ animals (**Figures 3C, D**). After antigen encounter from FDCs, B cells must obtain selection signals from T follicular helper (Tfh) cells. Insufficient signaling activates apoptosis or limits the number of subsequent B cell divisions. Therefore, lack of Tfh cells could be another explanation for the observed differences in GC formation. However, when analyzing the number of intrafollicular CD4^+^ cells by immunohistochemistry, no quantitative differences were found between genotypes (**Figure S4**).

### Changes in the splenic architecture of $s1pr_{4}^{–/–}$ animals did not result in an altered early antibody response after sepsis induction

**Figure 4:**
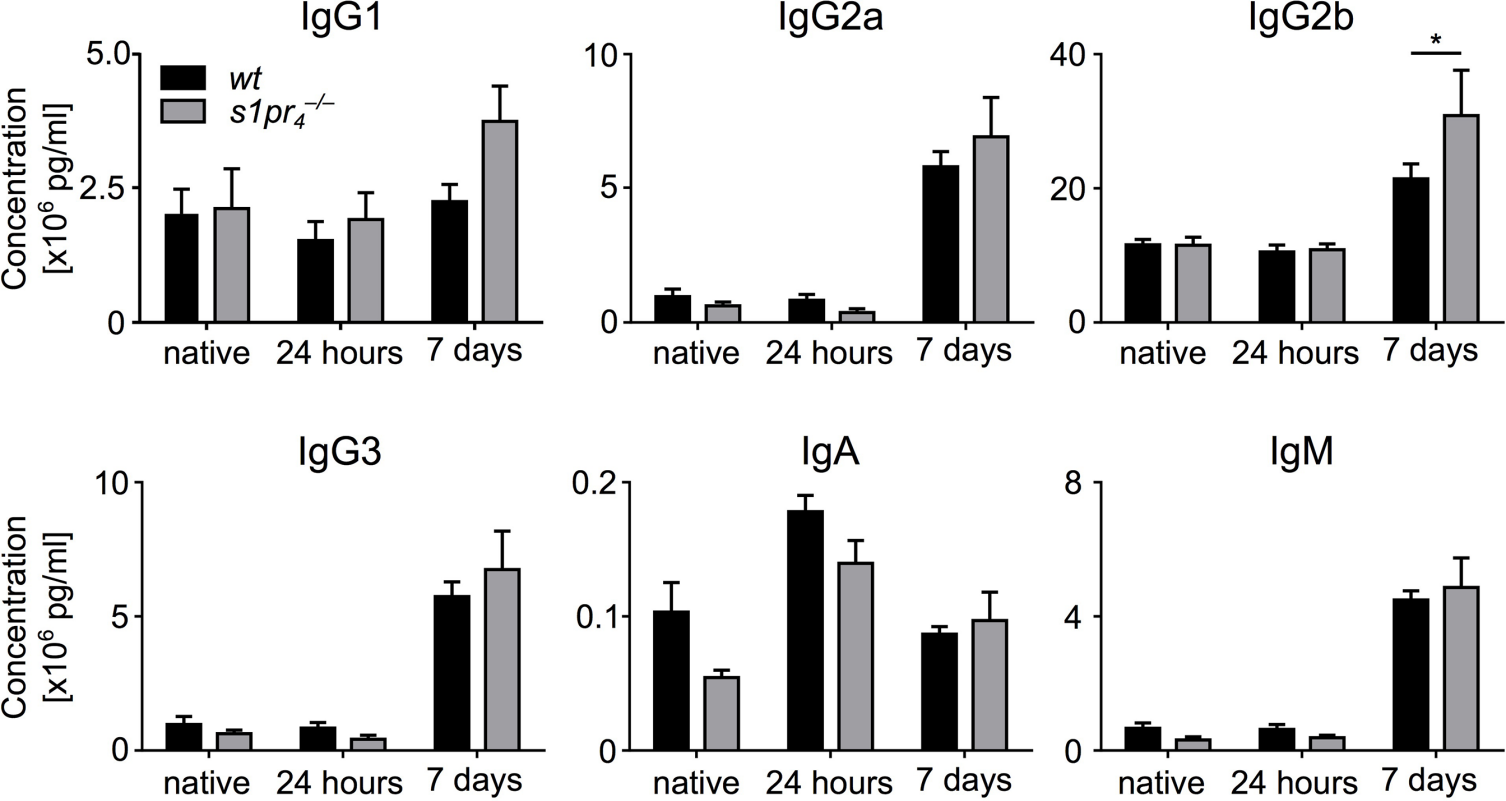
Dynamics of plasma immunoglobulin levels after sepsis induction. Blood plasma of wildtype (*wt*) and S1PR_4_-deficient ( $s1pr_{4}^{-/-}$ ) mice was analyzed for immunoglobulin levels. Three groups were compared: “native” (without sepsis induction), “24 hours” and “7 days” after sepsis induction *via* CASP. Means + SEM of n = 5 to 8 animals per group. *p < 0.05.

Given the differences between the size of GCs between the *wt* and the $s1pr_{4}^{-/-}$ genotype, we assessed antibody production 24 hours and 7 days after CASP induction. No differences in antibody levels of the IgG1, IgG2a, IgG3, IgM and IgA isotype could be detected between *wt* and $s1pr_{4}^{-/-}$ animals. IgG2b levels were slightly but significantly increased in $s1pr_{4}^{-/-}$ animals (**Figure 4**).

### Follicular shuttling of marginal zone B cells during abdominal sepsis is unchanged in $s1pr_{4}^{–/–}$ mice

**Figure 5:**
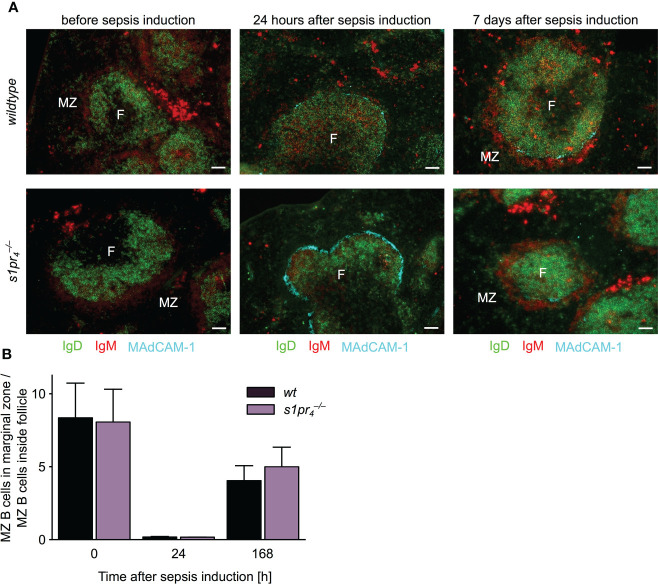
Follicular shuttling of MZ B cells in polymicrobial abdominal sepsis. **(A)** Spleens of mice before, one and seven days after sepsis induction were stained for marginal zone (MZ) B cells. MZ B cells were identified as IgD^–^ IgM^+^. Before sepsis induction, MZ B cells are located in a red IgM^+^ positive ring outside of green IgD^+^ follicles (F). Twenty-four hours after sepsis induction, most MZ B cells moved into the follicle and behind the turqoise MAdCAM-1^+^ marginal sinus. Seven days after the induction, MZ B cells oscillated back into the marginal zone as evidenced by the re-apparance of the perifollicular IgM^+^ cells. Representative images of n = 5 animals per group are shown. White scale bar equals 50 µm. **(B)** Quantitative analysis of MZ B cell shuttling during polymicrobial abdominal sepsis. The ratio represents the proportion of MZ B cells localized in the marginal zone to MZ B cells localized in the white pulp (n = 5; mean + standard deviation).

A B cell subpopulation with an unique migrational behavior are marginal zone (MZ) B cells, which shuttle antigens into the follicle. This marginal zone/follicular shuttling of MZ B cells is a prerequisite for efficient systemic antigen capture and delivery to FDCs. The process is tightly controlled by S1P signaling (27, 28). In order to test whether this process is affected by S1PR_4_-deficiency, the localization of MZ B cells was assessed before, one and seven days after sepsis induction (**Figure 5A**). Before sepsis induction, both $s1pr_{4}^{-/-}$ and *wt* MZ B cells were present at their usual localization in the follicle periphery. Twenty-four hours after sepsis induction, the proportion of MZ B cells located within the follicle increased. This process occurred to a similar extent in $s1pr_{4}^{-/-}$ and *wt* animals. On the seventh day after sepsis induction, the majority of MZ B cells had relocated back into the marginal zone in both genotypes. No quantitative differences in follicular shuttling of MZ B cells could be detected between $s1pr_{4}^{-/-}$ and *wt* animals (**Figure 5B**).

### T cell dependent IgM but not IgG humoral immune response is affected by S1PR_4_ deficiency

**Figure 6:**
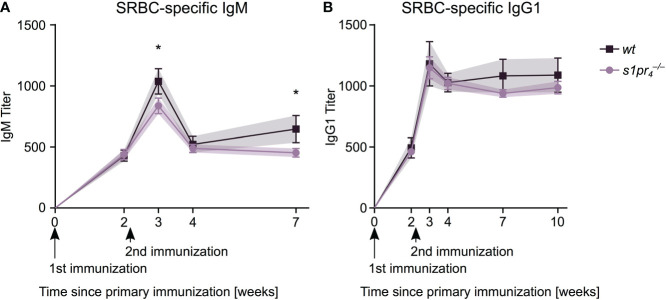
SRBC-specific antibody response after intraperitoneal immunization. Mice of both genotypes were immunized by intraperitoneal injection of sheep red blood cells (SRBC) at days zero and fourteen. SRBC-specific antibody response was analyzed after retro-orbital puncture at days 0, 14, 21, 28 and 49 for IgM **(A)** and additionally at day 70 for IgG1 **(B)**. Data shown for n = 5 animals per genotype. Values represent mean ± SD. * *p*< 0.05.

The sepsis induced early antibody response – which is predominantly T cell independent (23) – was not affected by S1PR_4_ deficiency. We hypothesized that a T cell dependent immune response that relies on precise B-cell-T-cell interactions within the germinal center may be influenced by the lack of S1PR_4_-mediated signaling. Mice were immunized with sheep red blood cells (SRBCs), a T cell dependent (TD) antigen known to induce a strong GC reaction. The primary IgM response to SRBC was similar in $s1pr_{4}^{-/-}$ and *wt* animals, 14 days after the primary immunization (**Figure 6A**). However, one week after secondary immunization, IgM levels were significantly reduced in $s1pr_{4}^{-/-}$ animals compared to *wt* animals, a difference that was also present five weeks after secondary immunization. The IgG1 response to the primary and secondary immunization showed no statistically significant differences in $s1pr_{4}^{-/-}$ and *wt* animals (**Figure 6B**).

## Discussion

Follicular B cells and MZ B cells express high levels of S1PR_1_ and lower levels of S1PR_3_ and S1PR_4_, while S1PR_2_ expression is very low and S1PR_5_ expression absent (4, 29). Compared to follicular B cells, S1PR_2_ expression is up-regulated in germinal center B cells (7). While distinct functions have been attributed to the S1P receptors S1PR_1_ – S1PR_3_ in splenic follicular B cells and MZ B cell biology, the role of S1PR_4_-mediated signaling in the spleen remains obscure (7, 27, 30).

In the present manuscript, we show that the size of splenic primary follicles was significantly reduced in $s1pr_{4}^{-/-}$ mice compared to *wt* animals, while the cumulative size of the B220^+^ B cell area was similar in both strains. Interestingly, mice deficient for the chemokine receptor CXCR5 also show structural aberrances of primary follicle structure (31, 32). Adoptively transferred CXCR5-deficient B cells fail to enter the splenic primary follicle (31). We have previously shown that, although S1PR_4_-mediated signaling induces chemotactic migration in peritoneal B1a and B1B cells, splenic B cells show no chemotactic response to S1P gradients (33, 34). We therefore examined whether S1PR_4_-mediated signaling modifies CXCR5-mediated chemotaxis. Indeed, S1PR_4_ deficiency resulted in a significantly reduced chemotactic response of splenic B cells to CXCL13 gradients, while S1P alone did not induce a chemotactic response in these cells. These results clearly indicate that S1PR_4_-mediated signaling modifies the CXCR5-mediated chemotactic response of follicular B cells, possibly resulting in the observed structural aberration in the spleen of $s1pr_{4}^{-/-}$ mice. Interestingly, interference with CXCL12- and CXCL13-induced chemotaxis has also been shown for another S1P receptor, S1PR_2_, in germinal center B cells *in vitro* (7). S1PR_2_-mediated signaling negatively modified CXCL13-induced chemotaxis, while S1PR_4_ increased CXCL13-induced chemotaxis in splenic B cells in our experimental setting (7). These data suggest that modulation of CXC-chemokine induced chemotaxis is a common mechanism in the S1P-mediated control of B cell migration.

Transitional type 1 B cells are the link between the bone marrow and the spleen, where they represent the initial B cell immigrants (35, 36). Interestingly, inhibition of S1P-mediated signaling has been shown to increase transitional B cell numbers in the peripheral blood of multiple sclerosis patients (37). In our S1PR_4_ deficient mouse model the number of transitional B cells in the spleen is significantly reduced. The mechanisms regulating the immigration of transitional B cells from blood to the spleen are not well understood. Our results suggest that S1PR_4_ may be involved in this process, and its deficiency may result in increased peripheral transitional B cell numbers by inhibiting entry into the spleen. Further research is required in order to verify this hypothesis.

Numbers of pro-/pre-B cells are reduced in the bone marrow of $s1pr_{4}^{-/-}$ animals. CXCL12 signaling is required for the development of pre-pro-B and pro-B cells, probably by regulating the positioning of these cells in their appropriate niches within the bone marrow (38). B cell precursors are significantly reduced in bone marrow of chimeric wildtype mice that have been reconstituted with CXCR4 deficient fetal cells (39). Moreover, pre-B cells were increased in the blood of CXCR4 deficient animals, indicating that the retention of B cell precursors within the bone marrow is dependent on CXCR4 (40). In splenic T cells, S1P has been shown to act in synergy with CXCL12 (41). Further experiments should investigate whether similar mechanisms are involved in the reduction of pre-/pro-B cell numbers in $s1pr_{4}^{-/-}$ mice.

Marginal zone (MZ) B cells physiologically shuttle between the MZ and the follicle, thus ensuring the effective transfer of antigens between these two localizations (4, 8). Only about 55% of MZ B cells are localized in the MZ, while 45% can be found inside the follicle (27). The respective proportions are determined by the expression levels of S1PR_1_ and CXCR5 (27). But also S1PR_3_-mediated signaling has been shown to impact MZ B cell shuttling (27). The data shown in this manuscript demonstrates that S1PR_4_-mediated signaling does not affect the proportion of MZ B cells in the MZ and the follicular area. However, it remains to be investigated whether the exchange rate, which has been shown to reach 20% per hour (27), is affected by S1P signaling *via* S1PR_4_. *In vitro* imaging in the intact spleen in our S1PR_4_ deficient mouse model will answer this question in the future. At the same time, total MZ B cell numbers were significantly increased in naïve $s1pr_{4}^{-/-}$ animals the FACS analysis of the whole spleen. Whether these changes are the result of altered migrational processes, an impact on the proliferative capacity of MZ B cells or MZ B cell survival remains to be shown in further experiments. S1PR_4_ has been shown to affect cell proliferation in S1PR_4_ overexpressing T cell lines (42). Experiments performed in our own lab showed no influence of S1PR_4_ mediated signaling on the proliferation of peritoneal B cells (data not shown). Further research is required to clarify the role of S1PR_4_ mediated signaling in MZ B cell proliferation and survival.

Only moderate micro-anatomical differences exist in $s1pr_{4}^{-/-}$ animals under steady-state-conditions and we wondered whether strong B cell activation would aggravate these histopathological changes. Abdominal sepsis is a clinically relevant disease state resulting in such a strong B cell activation (23). While the primary T cell dependent B cell response against specific antigens is severely impaired in septic mice, a strong unspecific increase of serum IgM and IgG levels as well as a pronounced germinal center reaction within the spleen can be observed in murine models of sepsis (24, 43). The main source of the IgM and IgG secreting cells in this model was the spleen (23). In the murine CASP model used herein, S1PR_4_ deficiency resulted in a significantly reduced GC formation in the septic spleen. Participation of S1P-mediated signaling in the development of the germinal center response has been previously reported by Han et al., who observed a reduced germinal center formation in mice after fingolimod treatment (12). However, the identity of the receptors mediating this effect has not yet been dissected. Disorganized development of GCs in the spleen has also been reported in the absence of CXCR4 expression in B cells (31, 44, 45). Both CXCL12 and CXCL13 have been implicated in the regulation of GC organization in dark and light zone (45). The histopathological features of $s1pr_{4}^{-/-}$ spleens are reminiscent of those seen in mice with a B cell specific conditional CDC42 knock-out, showing GCs of reduced size (46). CDC42 is part of the S1PR_4_ associated intracellular signaling cascade in lymphocytes (47). According to the recycling hypothesis, the development of an effective GC reaction depends on multiple rounds of B cell circulation between light and dark zone of the GC (25). These iterative rounds of mutation and selection in their respective zones are necessary for a stepwise optimization of B cell affinities towards the locally presented antigen, giving rise to affinity-matured memory and plasma cells (26). Our findings suggest that S1PR_4_ impacts the chemotactic control of B cell circulation resulting in structural aberrances of GC development in $s1pr_{4}^{-/-}$ animals. However, further experiments are required to examine the hypothesis that S1PR_4_ mediated signaling modulates not only CXCL13 induced chemotaxis but also that of other chemokines, e.g. CXCL12.

Both FDC and Tfh cells are essential for the development of an effective GC reaction (26, 48). Avancena et al. reported that a predetermined number of FDC clusters determine the maximum number of GCs that may develop after antigenic stimulation. Our results clearly show that the number of FDC clusters is similar in *wt* and $s1pr_{4}^{-/-}$ animals, thus excluding an influence of S1PR_4_ deficiency on the number of FDC clusters. However, since the size of FDC clusters was significantly reduced in $s1pr_{4}^{-/-}$ animals, further experiments involving conditional knock-out models of S1PR_4_ in either B cells or FDC are required to determine the respective contribution of S1PR_4_ expression in these two cell types for the development of the GC reaction. Tfh cells are responsible for both initiation and maintenance of the GC reaction. Their numbers showed no differences in $s1pr_{4}^{-/-}$ and *wt* animals, therefore excluding an influence of S1PR_4_ deficiency on Tfh accumulation as the source for the reduced GC size in the spleens of septic $s1pr_{4}^{-/-}$ animals.

Although the $s1pr_{4}^{-/-}$ genotype is characterized by a compromised development of splenic GCs, antibody production after polymicrobial intra-abdominal challenge resulted in only minor differences between $s1pr_{4}^{-/-}$ and *wt* animals. In CASP-induced sepsis, the spleen is the main source of antibody production (23). However, CXCR5 deficient mice also display a compelling disturbance of GC formation, while antibody formation upon immunization with T cell dependent antigens is normal (31). In our model, it remains to be determined whether normal antibody levels in $s1pr_{4}^{-/-}$ animals are due to extrasplenic antibody formation or whether the formation of these predominantly low-affinity antibodies is mainly due to the activity of short-lived plasma cells and not due to affinity maturated plasma cells originating in the splenic follicles (49, 50). In contrast, after immunization with SRBC, production of SRBC-specific IgM antibodies was slightly but significantly reduced in $s1pr_{4}^{-/-}$ animals after the second immunization cycle. This reflects the disturbance of T cell dependent antibody production in $s1pr_{4}^{-/-}$ animals, which requires specific interaction between B and T cells. The implication of S1P signaling in the development of an effective GC response and the production of high affinity antibodies has been previously elegantly revealed using the functional S1P antagonist fingolimod (12). Fingolimod binds to four out of five S1PRs (S1PR_1_, S1PR_3_ – S1PR_5_). However, since fingolimod is an unspecific inhibitor of S1P signaling, it remains to be shown whether the morphological changes in GC development secondary to S1PR_4_ deficiency affect also the affinity maturation during the antibody response

The S1P-S1PR axis is a complex signaling system. Cells usually express several S1PRs on their surface. The difference in signaling through various S1PR resides in variations of G-protein coupling of the different S1PRs (51). B cells express S1PR_1_ and S1PR_3–4_, GC B cells additionally S1PR_2_ (4, 7, 29). All of these receptors share down-stream signaling *via* G_i_ proteins (51). Thus, we cannot formally exclude that some effects of S1PR_4_ deficiency may be masked by compensatory signaling *via* other S1P receptors. Further experiments using receptor specific antagonists *in vitro* are required to exclude this possibility. In order to better differentiate B cell intrinsic from B cell extrinsic effects of S1PR_4_ mediated signaling and their mechanistic implication in the generation of the morphological and functional alterations reported in this manuscript, further experiments using conditional B cell specific S1PR_4_ knock-out models should be performed.

In summary, S1PR_4_-deficient mice exhibit a regular lymphatic architecture with only slight alterations in follicle size. During systemic infectious challenge reduced germinal center formation was observed. However, this did not translate into a relevant impairment of RBC specific antibody formation in our model. These results indicate a role for S1PR_4_ expression during the development of the GC reaction and warrant further research into consequences of impaired S1PR_4_ signaling.

## Data availability statement

The raw data supporting the conclusions of this article will be made available by the authors, without undue reservation.

## Ethics statement

The animal study was reviewed and approved by Landesamt für Landwirtschaft, Lebensmittelsicherheit u. Fischerei Mecklenburg Vorpommern (LALLF M-V) and by the Landesamt für Gesundheit und Soziales Berlin (LAGeSo). Written informed consent was obtained from the owners for the participation of their animals in this study.

## Author contributions

Conceptualization: JR and TS. Methodology: TS, AK and JR. All authors partook in the experiments. Software: JR, AK. Validation: JR and TS. Formal analysis: JR and TS. Writing original draft: JR. Writing review and editing: TS, AK, CH, MH and FL. Visualization: JR. Supervision: TS. All authors contributed to the article and approved the submitted version.

## Funding

This work was financed by an University Research Fund (Forschung und Lehre) of the Faculty of Medicine of the University of Greifswald. J.R., M.H. and F.L. were financially supported by a scholarship from the Gerhard Domagk programe of the University Medicine Greifswald.

## Acknowledgments

We would like to thank Antje Janetzko for dedicated technical and logistical support.

## Conflict of interest

The authors declare that the research was conducted in the absence of any commercial or financial relationships that could be construed as a potential conflict of interest.

## Publisher’s note

All claims expressed in this article are solely those of the authors and do not necessarily represent those of their affiliated organizations, or those of the publisher, the editors and the reviewers. Any product that may be evaluated in this article, or claim that may be made by its manufacturer, is not guaranteed or endorsed by the publisher.

## Abbreviations

BCR, B cell receptor; CASP, colon ascendens stent peritonitis; DAPI, 4’,6-diamidino-2-phenylindole; ELISA, enzyme linked immunosorbent assay; FACS, fluorescence-activated cell sorting; FDC, follicular dendritic cell; FO, follicular (B cell); GC, germinal center; MZ, marginal zone; PALS, periarteriolar lymphoid sheaths; PNA, peanut agglutinin; S1P, sphingosine-1-phosphate; S1PR, S1P receptor; $s1pr_{4}^{-/-}$ , mouse homozygous deficient in S1PR_4_ on a C57BL/6J background; SRBC, sheep red blood cell; Tfh, follicular T helper cell; *wt*, wild-type (C57BL/6J) mouse.
