# Supplementary material for: S1PR4 deficiency results in reduced germinal center formation but only marginally affects antibody production
Source: Front Immunol. 2022 Dec 2;13:1053490. doi: 10.3389/fimmu.2022.1053490 (PMC9755867; doi:10.3389/fimmu.2022.1053490)
Supplement: Supplementary file 1 [file DataSheet_1.pdf]

Supplementary material: S1PR4 and germinal centers

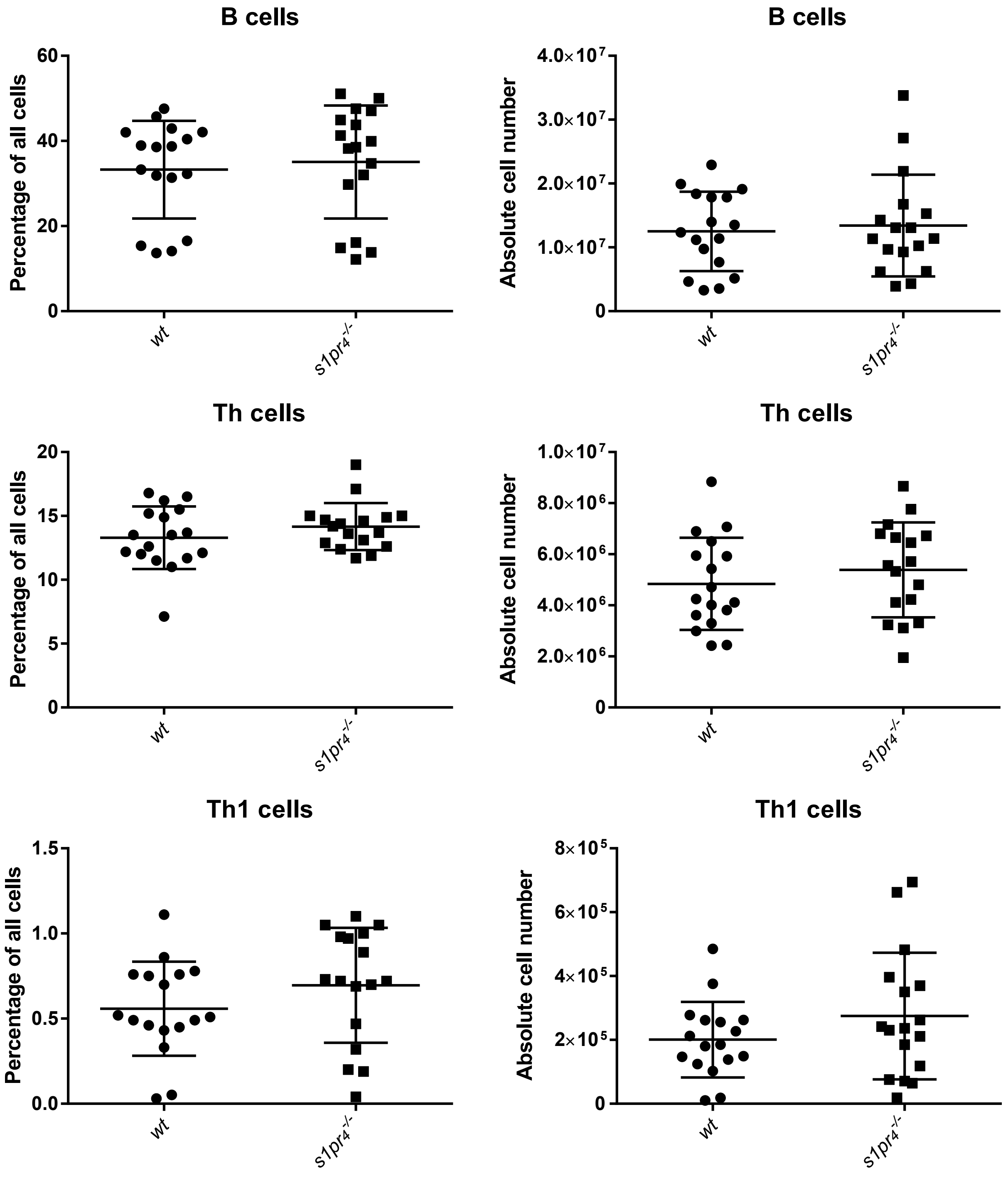

Supplemental Figure S1: Flow cytometric analysis of splenocytes from $S1PR_4$-deficient mice.

Single cell suspensions of 1 x 10^6 splenocytes were analyzed by flow cytometry. Absolute cell numbers were calculated using TruCount™ Beads (BD Biosciences). Cell populations were identified as: B cells (CD19+ CD3–), T cells (CD19– CD3+), Th cells (CD3+ CD4+), Th1 cells (CD3+ CD4+ T-bet+), Th2 cells (CD3+ CD4+ GATA3+), Th17 cells (CD3+ CD4+ RORγt+) and Tregs (CD3+ CD4+ FoxP3+). Mean ± standard deviation of n = 17 animals per genotype.

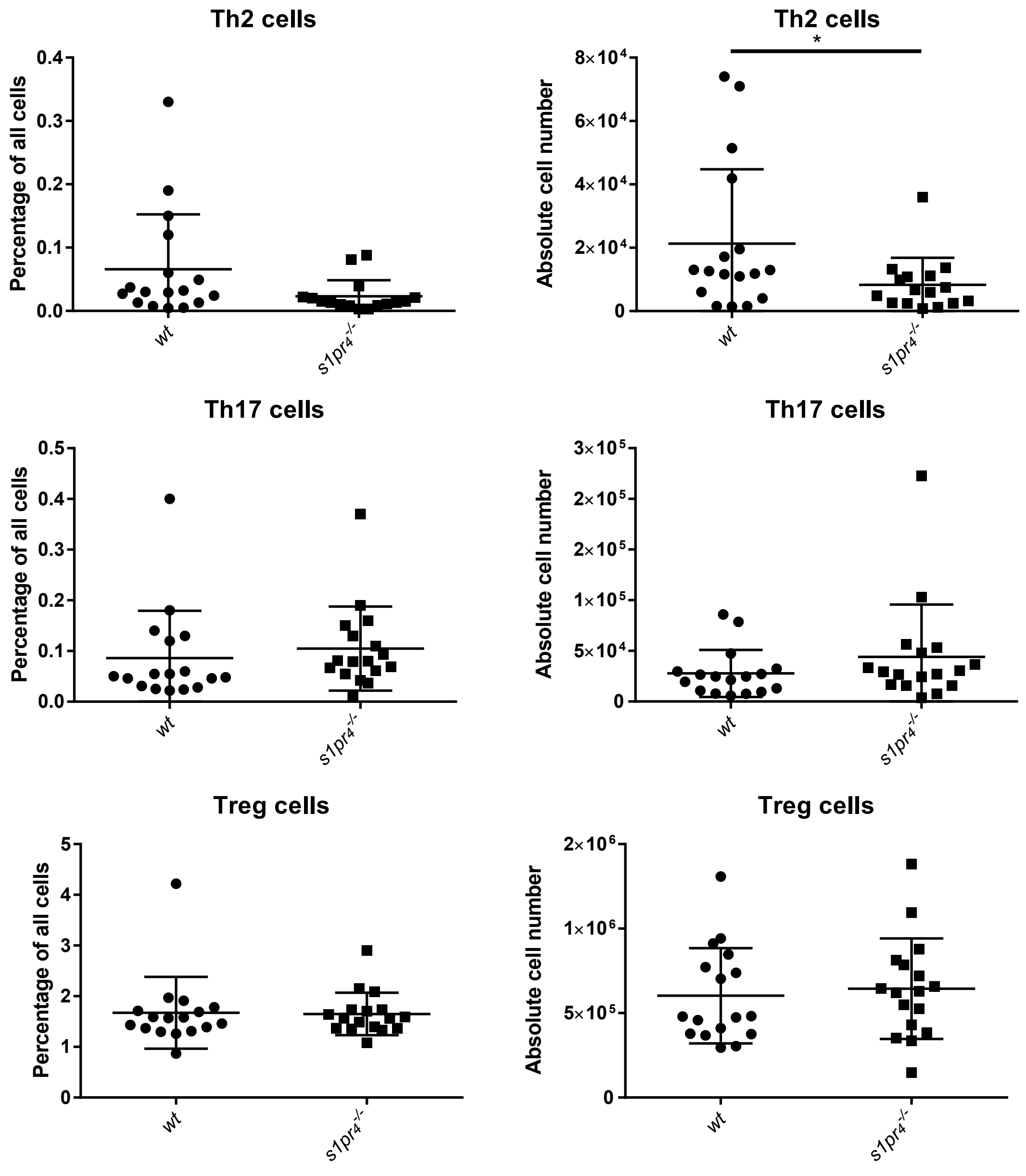

Supplemental Figure S1: Flow cytometric analysis of splenocytes from S1PR$_4$-deficient mice. ***(continued)***

Single cell suspensions of 1 x 10^6 splenocytes were analyzed by flow cytometry. Absolute cell numbers were calculated using TruCount™ Beads (BD Biosciences). Cell populations were identified as: B cells (CD19+ CD3–), T cells (CD19– CD3+), Th cells (CD3+ CD4+), Th1 cells (CD3+ CD4+ T-bet+), Th2 cells (CD3+ CD4+ GATA3+), Th17 cells (CD3+ CD4+ RORγt+) and Tregs (CD3+ CD4+ FoxP3+). Mean ± standard deviation of n = 17 animals per genotype.

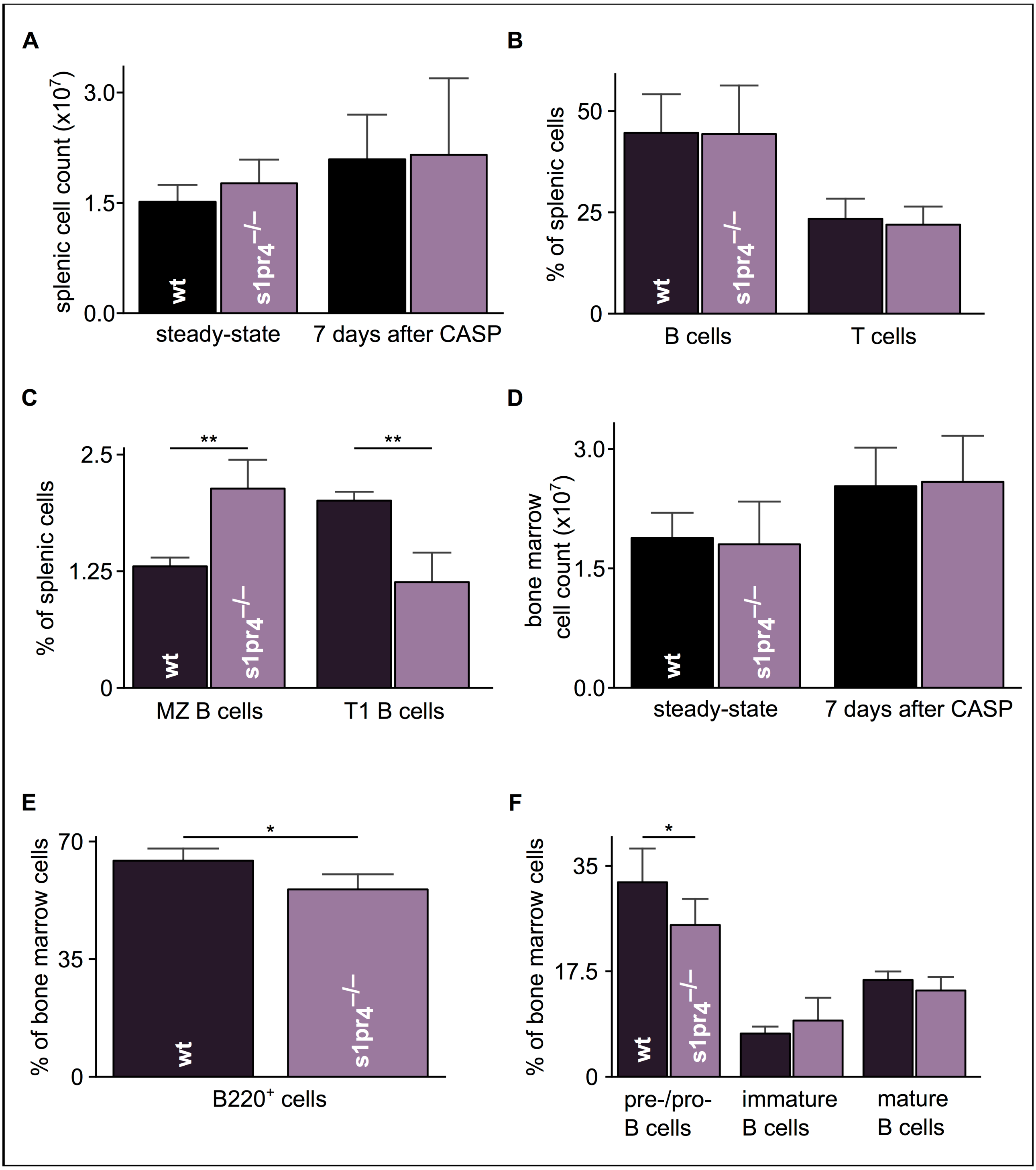

Supplemental Figure S2: Quantification of spleen and bone marrow B cell populations.

Flow cytometric analysis of spleen **(A-C)** and bone marrow **(D-F)** B cells populations from wildtype (wt) and $S1PR_4$-deficient ($s1pr_4^{-/-}$) mice. **A:** Total splenic cell count measured in spleen of both genotypes. **B:** Spenic B cells were identified as $CD19^+$ and T cells as $CD3^+$ cells. **C:** Splenic marginalzone (MZ) B cells were identified as $CD21^{hi}$, $CD23^+$, IgM^{hi}; and transitional type 1 (T1) B cells as $CD21^{lo}$, $CD23^{hi}$, IgM^{hi}. **D:** Total bone marrow cell count of both genotypes. **E:** B220-positive cells in the bone marrow. **F:** Bone marrow B cells were identified as pre-/pro-B cells ($B220^+$ IgM^-), immature B cells ($B220^{int}$ IgM^+) and mature B cells ($B220^{hi}$ IgM^+). Values represent mean + standard deviation of $n = 6$ animals per genotype. * $p < 0.05$, ** $p < 0{,}01$.

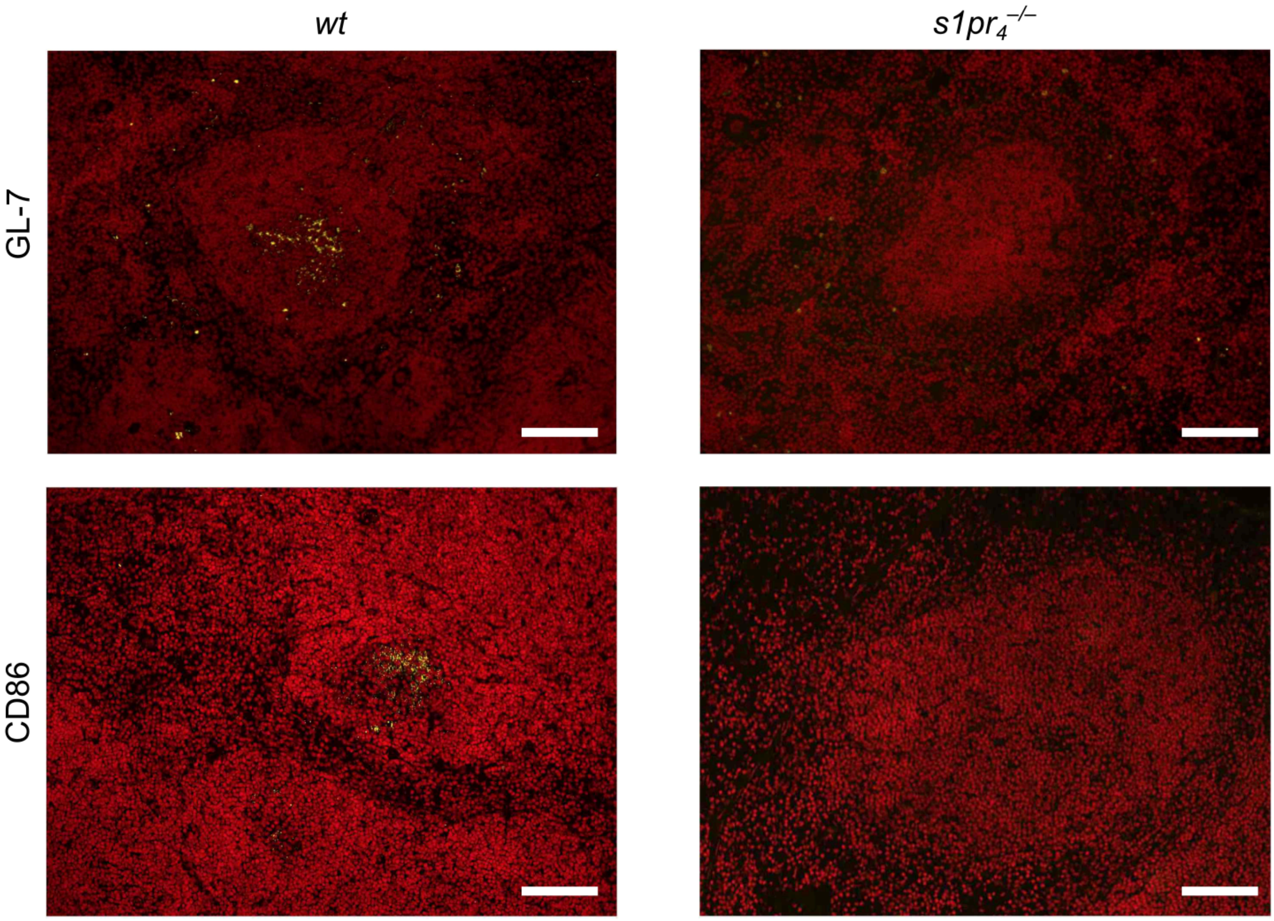

Supplementary figure S3: Staining of germinal centers using GL-7 and anti-CD86.

After sepsis induction via colon-ascendens stent peritonitis (CASP), mice were sacrificed and splenic sections were stained using Draq5™ (red) for nuclei, anti-B220 (not shown) and GL-7 or anti-CD86 (yellow) for identification of GC B cells. White bars equal 100 µm. Representative for n = 5 animals per conditions.

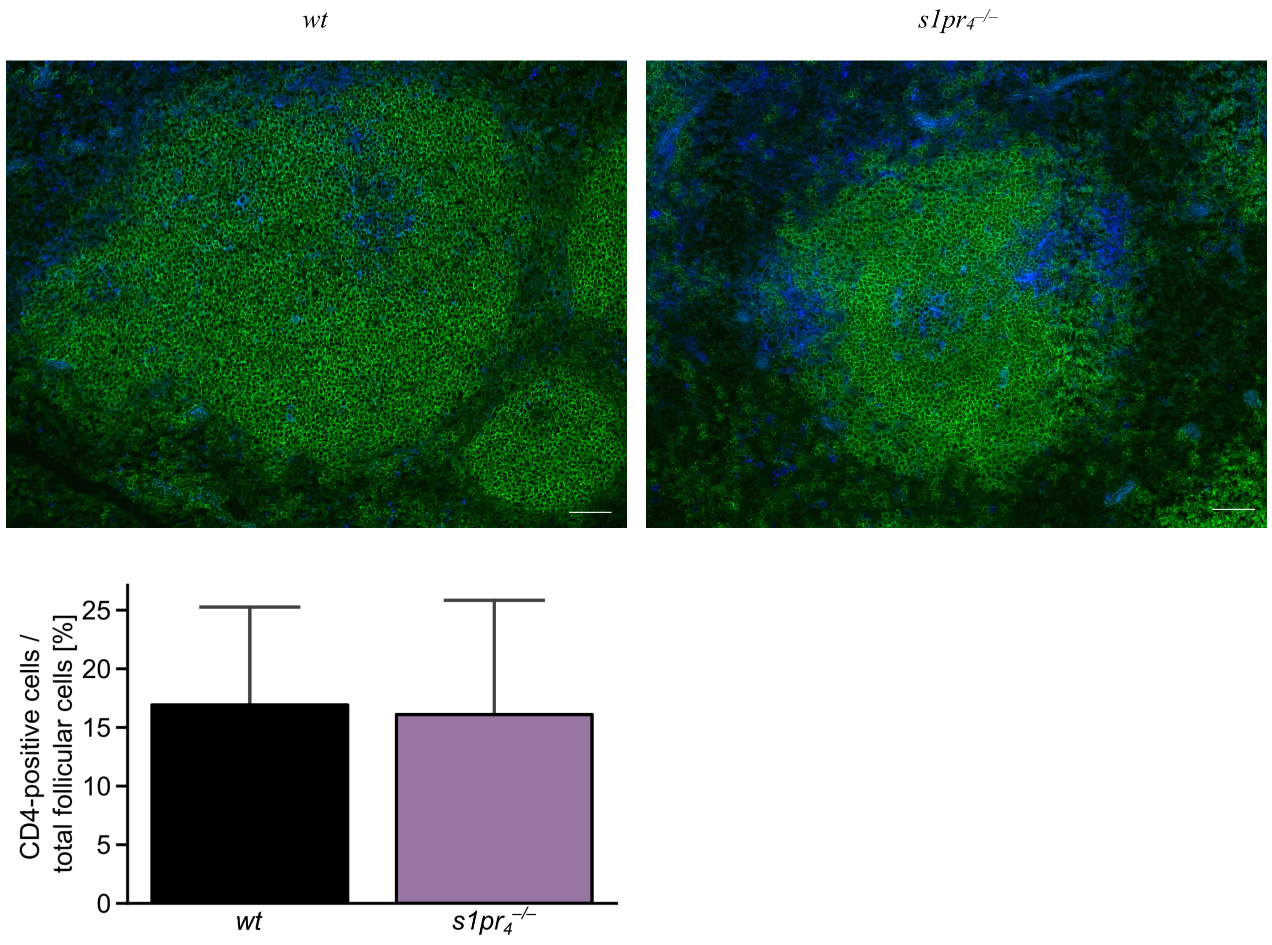

Supplementary figure S4: Quantification of intrafollicular CD4-positive cells.

Splenic sections were stained with anti-B220 (green), anti-CD4 (blue) and Draq5™ for nuclei detection (not shown). Cells of interest were identified as B220– CD4+ cells within the follicle. Follicle border points were identified using Jarvis' convex hull algorithm. Follicle border was continuously computed via Fourier transformation. Cell centroids within follicle borders were analyzed using the cell classification algorithm of QuPath. Values represent mean + standard deviation of n = 5 animals per genotype. White scale bar equals 50 µm. No significant ($p > 0.05$) difference was found between genotypes.
